# The Activity of Neutral α-Glucosidase and Selected Biochemical Parameters in the Annual Cycle of Breeding Carp (*Cyprinus carpio* L.)

**DOI:** 10.1371/journal.pone.0142227

**Published:** 2015-11-11

**Authors:** Julia Kotońska-Feiga, Wojciech Dobicki, Przemysław Pokorny, Wojciech Nowacki

**Affiliations:** 1 Department of Hydrobiology and Aquaculture, Institute of Biology, Wrocław University of Environmental and Life Science, Wrocław, Poland; 2 Department of Immunology, Pathophysiology and Veterinary Preventive Medicine, Wrocxaw University of Environmental and Life Science, Wrocław, Poland; Karlsruhe Institute of Technology, GERMANY

## Abstract

The aim of the study was to demonstrate seasonal changes in the hydrolytic and transferase activity of neutral α-glucosidase, the level of glucose, cholesterol, triglycerides and total protein in the annual breeding cycle of the carp. The study was conducted on fish from a fish farm in Lower Silesia (Poland). Blood serum was collected from the heart in: June, September and December of two consecutive years. The results of the study show that the hydrolytic and transferase activity of neutral α-glucosidase, as well as the results of basic biochemical parameters are highest in summer, when the fish seek and intake food intensively. The lowest values were observed in spring, when carp have the lowest metabolism after the wintering period.

## Introduction

EC 3.2.1.x O-glycoside hydrolases are a widespread sub-subclass, of 178 enzymes [1]. Enzymes from this sub-subclass carry out a variety of functions depending on their subcellular location, as well as the type of organ and organism in which they occur. Among animal α-glucosidases the following are distinguished: acid and neutral α-glucosidase, microsomal α-glucosidases and complexes of α-glucosidase in the intestinal villi.

Neutral α-glucosidase (EC 3.2.1.20) has a hydrolytic and transferase activity. It catalyzes hydrolysis, releasing α D-glucose and acting on the non-reducing end of the molecule. Because neutral α-glucosidase belongs to the group of enzymes that preserve the same product conformation as the substrate. Therefore it is capable of catalyzing transferase reaction (transglycosylation).

Neutral α-glucosidase hydrolyzes the α-1,4 glycosidic bonds of maltooligosaccharides and maltodextrines derived from the hydrolysis of glycogen by α-amylases [2, 3]. Neutral α-glucosidase hydrolyzes α-1,2, α-1,3; and α-1,6 glycosidic bonds to a smaller extent [4]. At higher substrate concentrations the enzyme shows transferase activity, synthesizing oligosaccharides such as maltodextrins. In contrast to the lysosomal (acid) α-glucosidase, which is active at pH 3–6, the neutral α-glucosidase is active at pH 6–7 and is a cytoplasmic enzyme. It is widespread among microorganisms, plants, and animals. Its presence can be observed in numerous tissues and organs, as well as in the blood serum [5]. In recent years, several studies have focused on the activity of neutral α-glucosidase in human semen and its usefulness in evaluating the semen quality [6–12].

Hydrolytic activity of neutral α-glucosidase in the blood of mammals was studied by Chiba [4], who demonstrated significant species-specific differences–from 1.3 mIU cm^-3^ in human serum to 24500 mIU cm^-3^ in pig serum. Other studies involved the transferase activity of neutral α-glucosidase [13, 14].

Fish differ from warm-blooded animals mainly due to their seasonal metabolic variation.This is particularly evident in, freshwater and farm fish living in ponds with variable water temperature owing to changing weather conditions and seasonality. This fact should be taken into account when determining the activity of enzymes in the body fluids, as well as in tissues of the fish. Carp is a fish whose breeding cycle covers all seasons in the European climate zone. When studying the activity of enzymes in the body fluids and tissues of these species, one should take into account the seasonal variability which may considerably affect the results. Few studies are available on the activity of the enzymes in fish blood with special focus on eventual seasonal changes. There are no literature reports of seasonal neutral α-glucosidase changes in fish blood serum.

The aim of the study was to evaluate the seasonal changes in the hydrolytic and transferase activity of neutral α-glucosidase and to determine the glucose, total protein, cholesterol and triglyceride levels in the serum of carp (*Cyprinus carpio* L.).

The obtained results may also be applicable to fish of a similar physiology and etiology, such as omnivorous thermophile fish, also exhibiting seasonal variability.

## Materials and Methods

The fishes were obtained from a commercial fishing farm–no specific permissions were required. The study also had no impact on endangered or protected species.

The study was approved by the II Local Ethical Committee for Research on Animals in Wrocław (II LKE). The sampling procedure and all experimental manipulations were reviewed and approved prior to obtaining the field permit.

The study was conducted over two consecutive years, during three distinctive seasons in the physiology and metabolism of carp–spring (the last decade of April), summer (September) and winter (December). Each year and each season, the carp were divided into two groups. The first group consisted of carp of lower weight–up to 1100 g (small carp); the second group carp of higher body weight (large carp). The animals were divided into two weight -groups in order to assess whether there is a correlation between the studied parameters and the fish body weight.

The animals were divided into 12 subgroups (5 specimens in each subgroup), depending on the weight of the fish, the season and year of study. Subgroup designations are shown in Table 1.

**Table 1:** Designation of experimental groups.

	1^st^ year			2^nd^ year		
Fish weight	Spring	Summer	Winter	Spring	Summer	Winter
**Small (< 1100 g)**	I	II	III	IV	V	VI
**Large (> 1100 g)**	VII	VIII	IX	X	XI	XII

After harvesting from the pond, the three-year-old carp were measured with the accuracy of 1 mm and weighed on an electronic scale with the accuracy of ±5 g.

No anesthetics were used during the study and a 2–4 ml blood sample was collected from the heart (by cardiac puncture) [15].

The sampled serum was centrifuged (Sigma 3 K 18) for 5 minutes at 4°C and 1000–1200 × g. A 0.5 ml sample was used for basic and enzymatic tests.

### Analytical methods

The concentration of glucose, cholesterol and triglycerides was determined through an enzymatic reaction using the Cormay Liquick Cor–GLUCOSE 60 kit, the Liquick Cor–CHOL 30 kit and the Liquick Cor–TG 30 kit (Poland). Total protein was determined using the Lowry method [16].

The hydrolytic activity of neutral α-glucosidase was determined as follows:

10 μl of serum was incubated at 30°C for 10 minutes with 90 μl of a 100 mM pH 6.8 phosphate buffer at containing 1.2 mg of maltose. The reaction was stopped by adding 200 μl of a 6% solution of trichloroacetic acid (TCA). The precipitated protein was centrifuged for 5 minutes at 10,000 × g. 50 μl of the supernatant was sampled and assayed for glucose content using the enzymatic method. To prepare the control sample the serum was added to a substrate solution containing 200 μl of TCA. The hydrolytic activity was expressed in IU cm^-3^ of serum.

The transferase activity of neutral α-glucosidase was determined as follows:

10 ml serum was incubated for 20 minutes at 30°C with 90 μl of a 100 mM pH 7.0 phosphate buffer at containing the p-nitrophenyl-N-maltoside (pNGlc2) substrate at a concentration of 40 mM. After thermal (100°C) interruption of the reaction and centrifugation of the sample for 5 minutes at 10,000×g, the supernatant was diluted 500-fold with redistilled water for HPLC and 80 μl of the product solution was injected to a HPX 87K column (Bio-Rad, USA). The amount of the resulting products was identified based on the retention time and was calculated by measuring the surface area of the separated products by dedicated software included in the Waters HPLC unit. Beforehand, a series of standard concentrations of the individual p-nitrophenyl-N-glycosides eluted through the column was prepared in order to determine the relationship between the surface area of the separation of the products and their concentration. The activity was expressed in μ-moles of maltooligosaccharides produced over a period of 1 minute at 30°C by the enzyme present in 1 ml of serum.

### Statistical analysis

Statistical analysis was performed using the Statsoft–Statistica 9.0 software. A three-way analysis of variance (ANOVA) and Tukey post hoc test were used to compare equal groups. Statistically significant differences were written in lower-case letters at p≤0.05 and with capital letters at p≤0.01.

Furthermore, an arithmetic mean (*x*) and standard deviation (SD) was calculated for each parameter.

## Results

The lowest hydrolytic activity occurred in the spring in both small and large carp (Table 2). This activity was roughly 1.5- fold higher in the winter and 2.5- fold higher in the summer.

**Table 2:** Seasonal activity of neutral α-glucosidase.

			Small carp			Large carp		
Parameter	Year		Spring	Summer	Winter	Spring	Summer	Winter
**Hydrolytic activity [IU cm** ^**-3**^ **]**								
	1^st^	*x*	0.84	2.22	1.22	1.07	2.45	1.43
		SD	0.24	0.13	0.25	0.29	0.31	0.17
	2^nd^	*x*	0.89	2.37	1.39	0.99	2.51	1.42
		SD	0.15	0.29	0.27	0.18	0.39	0.21
	All	*x*	0.87	2.30	1.31	1.03	2.48	1.43
		SD	0.20	0.21	0.26	0.24	0.35	0.19
**Transferase activity [IU cm** ^**-3**^ **]**								
	1^st^	*x*	1.75	5.24	2.49	2.16	5.58	3.01
		SD	0.36	0.24	0.49	0.43	0.40	0.46
	2^nd^	*x*	1.88	4.80	2.73	2.07	5.22	2.93
		SD	0.35	0.52	0.55	0.36	0.35	0.53
	All	*x*	1.82	5.02	2.61	2.12	5.40	2.97
		SD	0.36	0.38	0.52	0.40	0.38	0.50

*x*—arithmetic mean, SD–standard deviation

Very similar results were observed for seasonal differences in transferase activity of the neutral α-glucosidase under investigation. Transferase activity on pNGlc2 is higher than hydrolytic activity on maltose in all the six subgroups. In terms of seasons, identical pattern for transferase activity was observed. The lowest activity within the individual weight groups of the fish was demonstrated in the spring. In summer, as in the case of hydrolytic activity, an over 2.5-fold increase in transglycosylation occurred. In the winter, there was a significant decrease in transferase activity in small and large carp respectively (Table 2).

The study of the enzyme activity was carried out in two annual cycles, during which the average temperatures and amounts of sunlight were similar. No statistically significant differences were observed between the values of the studied parameters in the same seasons in the two consecutive years (Table 2); therefore, the quantitative data regarding the studied activities–both the mean (*x*) and the standard deviation (SD)–apply to measurements performed in both years.

Variations in both the hydrolytic activity and the transferase activity of neutral α-glucosidase occurring between seasons in the serum of carp are highly statistically significant p≤0.01.

Table 3 show the mean values of the levels of the glucose, total cholesterol, triglycerides and total protein in the blood serum of carp in all the experimental groups.

**Table 3:** Seasonal changes in fish size, glucose, cholesterol, triglycerides, and total protein concentrations.

		Small carp			Large carp		
Parameter		Spring	Summer	Winter	Spring	Summer	Winter
**Body length [cm]**	*x*	35.76	37.13	37.20	36.38	39.21	38.89
	SD	0.69	0.92	1.05	0.79	2.04	1.83
**Body weight [g]**	*x*	823.50	1039.00	999.50	1277.50	1694.50	1754.50
	SD	129.50	52.92	76.05	93.91	370.58	322.28
**Glucose [mg dL** ^**-1**^ **]**	*x*	76.69	151.75	82.56	76.36	164.94	87.96
	SD	23.49	19.36	17.07	22.31	33.04	13.81
**Cholesterol [mg dL** ^**-1**^ **]**	*x*	163.66	187.51	163.65	165.52	204.39	168.44
	SD	28.65	31.26	22.14	43.50	32.57	25.83
**Triglycerides [mg dL** ^**-1**^ **]**	*x*	125.07	169.12	151.48	122.34	189.40	171.30
	SD	39.73	29.03	35.76	30.62	32.81	28.26
**Total protein [%]**	*x*	1.97	2.75	2.11	1.97	2.94	2.26
	SD	0.08	0.35	0.17	0.04	0.26	0.13

*x*—arithmetic mean, SD–standard deviation

The activity of α-glucosidase in individual subgroups and groups with statistically significant differences are summarized in Table 4. The mean fish size as well as mean concentrations of total protein, cholesterol, glucose and triglycerides in the experimental subgroups and groups are presented in Table 5.

**Table 4:** Activity of neutral α-glucosidase in experimental subgroups and groups.

		Activity of neutral α-glucosidase	
	Specification	Hydrolytic[IU cm^-3^]	Transferase[IU cm^-3^]
**Subgroup**			
	I	0.84^A^	1.75^Aa^
	II	2.22^Bb^	5.24^C^
	III	1.22^A^	2.49^AB^
	IV	0.89^A^	1.88^Aa^
	V	2.37^B^	4.80^C^
	VI	1.39^Aa^	2.73^ABb^
	VII	1.07^A^	2.16^AB^
	VIII	2.45^B^	5.58^C^
	IX	1.43^Aa^	3.01^Bb^
	X	0.99^A^	2.07^ABa^
	XI	2.51^B^	5.22^C^
	XII	1.42^Aa^	2.93^Bb^
**Year**			
	1^st^ year	1.54	3.37
	2^nd^ year	1.59	3.27
**Season**			
	spring	0.95^A^	1.96^A^
	summer	2.39^C^	5.21^C^
	winter	1.36^B^	2.79^B^
**Size of the fish**			
	small	1.49^a^	3.15^A^
	large	1.65^b^	3.49^B^

Statistically significant differences: Different letters indicates differences AB—p ≤ 0.01; ab—p ≤ 0.05

**Table 5:** Levels of total protein, cholesterol, glucose, triglycerides as well as fish size in experimental subgroups and groups.

	Specification	Total protein [%]	Cholesterol [mg dL^-1^]	Glucose [mg dL^-1^]	Triglycerides [mg dL^-1^]	Body weight [g]	Body length [cm]
**Subgroup**							
	I	1.99^A^	163.7^a^	79.5^A^	127.6	854^A^	36.2^AB^
	II	2.63^BCb^	193.4	152.7^B^	165.5	1061^A^	37.1^AB^
	III	2.12^ABa^	145.2^A^	74.5^A^	155.9	963^A^	37.4^AB^
	IV	1.94^A^	163.6^a^	73.8^A^	122.6	793^Aa^	35.4^Aa^
	V	2.86^C^	181.6	150.8^B^	172.7	1017^A^	37.2^AB^
	VI	2.09^A^	182.1	90.6^A^	144.1	1036^A^	37.0^AB^
	VII	1.95^A^	172.0	69.1^A^	104.3^a^	1284^b^	36.4^AB^
	VIII	2.96^C^	173.3	155.0^B^	187.7^b^	1632^B^	38.5^BCb^
	IX	2.23^AB^	162.9^a^	86.5^A^	169.5	1808^Bc^	38.9^BC^
	X	1.98^A^	159.1^a^	83.6^A^	140.3	1271^b^	36.4^AB^
	XI	2.92^C^	235.5^Bb^	174.8^B^	191.1^b^	1757^Bc^	39.9^C^
	XII	2.28^AB^	174.0	89.4^A^	173.1	1701^B^	38.9^BC^
**Year**							
	1^st^ year	2.31	168.4	102.9	151.8	1267	37.4
	2^nd^ year	2.35	182.6	110.5	157.3	1262	37.5
**Season**							
	spring	1.97^A^	164.6^A^	76.5^A^	123.7^A^	1050^A^	36.1^A^
	summer	2.84^C^	196.0^Bb^	158.3^B^	179.3^B^	1367^B^	38.2^B^
	winter	2.18^B^	166.0^a^	85.3^A^	160.6^B^	1377^B^	38.0^B^
**Size of the fish**							
	small	2.27^a^	171.6	103.7	148.1	950^A^	36,7^A^
	large	2.39^b^	179.4	109.7	161.0	1575^B^	38,2^B^

Statistically significant differences: Different letters indicates differences AB—p ≤ 0.01; ab—p ≤ 0.05

SEM, p-values and interactions between factors have been attached in S1 and S2 Tables in the Supporting Information.

## Discussion

Both the hydrolytic and transferase activity of neutral α-glucosidase in the blood serum of carp vary significantly depending on the season of the annual breeding cycle.

A highly statistically significant increase in the level of glucose was found in the summer. This was associated with intensive food intake by the fish during this period. The increase in other parameters was also statistically significant in the summer with a tendency to persist during the winter. In spring, all the examined parameters remained at the lowest levels.

The low levels of glucose in the blood serum of carp harvested in the spring were caused by a low physiological activity of these fish in low water temperatures. At this time, the carp were beginning to intake food and increase their physical activity. The remaining studied biochemical parameters also reached their lowest concentrations during that period. It was not until an increase in water and air temperature to about 16–18°C that the carp began to seek and intake food intensively. Their physical condition and the intensity of metabolism increased. In summer, the concentration of serum glucose increased due to higher food intake and glycogen metabolism. In winter, when the fish ceased to feed, glucose levels decreased. In India, Jan et al. [15] found the lowest glucose levels in the blood of carp during winter and the highest serum glucose levels during autumn. However, climate differences between Poland and India should be taken into account.

The levels of cholesterol, triglycerides and total proteins in the blood serum of fish may serve as nutritional condition indices. As opposed to glucose, these parameters are not subject to rapid quantitative changes caused by short-term stress during blood collection. The increases in the concentrations of these three parameters observed during late summer were most likely the result of intense food intake and high activity of the fish at that time. This interpretation was confirmed by studies assessing the effects of different doses of food on the levels of triglycerides and cholesterol in the blood serum of carp [17]. Patriche et al. [18] studied chosen biochemical parameters in carp blood in two age groups and indicated higher values of glucose, protein, cholesterol and triglycerides in two-year-old carp compared to one-year-old carp.

Seasonal variations in the serum concentration of total protein in carp demonstrated in the presented results were also related to the intensity of metabolism. During winter and spring, when water temperatures are low and the fish case to intake food, the intensity of their metabolism, including that of the hepatopancreas which is a major source of serum proteins, is decreased. The renewal of serum proteins is slower when the metabolism is low. In the summer, there is increased protein synthesis within the body. The fish grow and gain weight. Thus, the total amount of protein in the serum significantly increased in this period.

Distinctive differences in the activity of lactate dehydrogenase (LDH) were found in Cyprynidae during different seasons. It was low during winter and spring and when compared to the cold seasons, it was almost three times higher during summer and twice as high during autumn [19].

By comparing the activity of aldolase and lactate dehydrogenase in muscles and blood serum of carp during summer and autumn (November), Melotti et al. [20] found that the serum activity of these enzymes was higher during autumn. However, they found the concentrations of these enzymes to decrease in autumn signifing reduced muscle metabolism. On the other hand, Kotoński and Sobiech [14] found a lower amylomaltase activity in the red and white muscles of carp during winter, when compared to that in summer.

Few studies on transferase activity of neutral α-glucosidase, with the objective of determining whether the measurement of this activity could have diagnostic significance in the future, have been conducted so far [21–23]. To date, the enzyme does not have clinical diagnostic value, which may be due to its very low activity in human blood. Since the demonstration of its activity in human semen and the location of the activity in the secretion of epididymis, the enzyme is used as an indicative parameter in the evaluation of semen [7, 9, 11, 24, 25]. Since 1999, neutral α-glucosidase is classified as a diagnostic enzyme, especially in the evaluation of semen parameters. To date this parameter has not been studied in the semen of fish. It may prove to be helpful in the evaluation of the quality of the semen with practical implications for aquaculture and fisheries. In addition, future research of neutral α-glucosidase in species that exhibit its high activity, especially in the blood, could prove that it is an important indicator for diagnostic purposes. Neutral α-glucosidase, which is active in the blood serum, shows very distinct species-specific quantitative variation [4]. This variation also concerns different species of fish (own unpublished observations).

The authors’ own research into the activity of neutral α-glucosidase in the blood serum of carp, as well as literature reports, imply that a number of factors must be considered in the interpretation of results when studying the activity of enzymes in fish. The most important ones include the species, season (especially in freshwater fish), stressors (that fish are exposed to and respond to accordingly), age, type of food and periods of feeding.

Most of the statistically significant differences of the concentrations of the studied parameters were demonstrated between individual subgroups and between seasons of the year (Table 4).

Hydrolytic and transferase activity, as well as the protein content showed highly statistically significant variation in all studied seasons (spring, summer, winter). Similarly, highly significant differences between spring and summer were also confirmed for total cholesterol, glucose and triglycerides. When comparing results obtained in summer and winter, a highly significant difference was observed for glucose and a statistically significant difference was observed for total cholesterol. The level of glucose, cholesterol, triglycerides and total protein in the blood serum of carp peaked during summer. In spring and winter, the levels of these parameters were statistically significantly lower. When comparing the results obtained in summer and winter, no statistically significant difference in the level of triglycerides was evident.

There were no statistically significant differences between the successive years of the study. It can therefore be assumed that the relationship between the studied parameters was not affected by small climate differences that undoubtedly occurred during the study period. In contrast, when comparing large carp with small carp, there were statistically significant differences in hydrolytic and transferase activity, as well as in the total protein concentration. This relationship proves that carp with higher weight (those growing faster and fed better) have higher enzyme activity. However, the level of total cholesterol, glucose and triglycerides in both size groups remained similar.

The effect of the following interactions was not confirmed statistically: year season, year size, season size, year season size.

## Conclusions

High hydrolytic and transferase activities of α-glucosidase, with distinct seasonal variations were found in the blood serum of carp.

The level of glucose, cholesterol, triglycerides and total protein in the blood serum of carp undergoes seasonal variations and peaks during summer. In spring and winter, the levels of these parameters were statistically significantly lower. This is associated with the water temperature, intensity of feeding of the carp and the condition of the fish. Weight may significantly affect the value of biochemical parameters in carp.

Lack of differences between successive years confirms the characteristic seasonal variation of the enzyme, irrespective of the weather within a single study year.

## Supporting Information

S1 Table Statistical informations for activity of neutral α-glucosidase in experimental subgroups and groups. (DOCX)

S2 Table Statistical informations for levels of total protein, cholesterol, glucose, triglycerides as well as fish size in experimental subgroups and groups. (DOCX)
