# Supplementary material for: The Activity of Neutral α-Glucosidase and Selected Biochemical Parameters in the Annual Cycle of Breeding Carp (Cyprinus carpio L.)
Source: PLoS One. 2015 Nov 11;10(11):e0142227. doi: 10.1371/journal.pone.0142227 (PMC4641633; doi:10.1371/journal.pone.0142227)
Supplement: S1 Table — (DOCX) [file pone.0142227.s001.docx]

**S1 Table. Statistical informations for activity of neutral α-glucosidase in experimental subgroups and groups.**

|  | **Specification** | **Activity of neutral α-glucosidase** | |
| --- | --- | --- | --- |
|  |  | **Hydrolytic**  **[IU·cm^-3^]** | **Transferase**  **[IU·cm^-3^]** |
| **SEM** | | 0.09 | 0.19 |
| **p-value** | **Subgroup** | 0.00 | 0.00 |
|  | **Year** | 0.41 | 0.36 |
|  | **Season** | 0.00 | 0.00 |
|  | **Size of the fish** | 0.02 | 0.00 |
| **Interaction** | **Year·Season** | 0.74 | 0.17 |
|  | **Year·Size** | 0.31 | 0.49 |
|  | **Season·Size** | 0.92 | 0.96 |
|  | **Year·Season·Size** | 0.96 | 0.75 |
