# Supplementary material for: The Activity of Neutral α-Glucosidase and Selected Biochemical Parameters in the Annual Cycle of Breeding Carp (Cyprinus carpio L.)
Source: PLoS One. 2015 Nov 11;10(11):e0142227. doi: 10.1371/journal.pone.0142227 (PMC4641633; doi:10.1371/journal.pone.0142227)
Supplement: S2 Table — (DOCX) [file pone.0142227.s002.docx]

**S2 Table. Statistical informations for levels of total protein, cholesterol, glucose, triglycerides as well as fish size in experimental subgroups and groups.**

|  | **Specification** | **Total protein**  **[%]** | **Cholesterol**  **[mg·dL^-1^]** | **Glucose**  **[mg·dL^-1^]** | **Tri-glycerides**  **[mg·dL^-1^]** | **Body weight**  **[g]** | **Body length**  **[cm]** |
| --- | --- | --- | --- | --- | --- | --- | --- |
|  |  |  |  |  |  |  |  |
| **SEM** | | 0.06 | 4.70 | 5.54 | 5.26 | 52.61 | 0.23 |
| **p-value** | **Subgroup** | 0.00 | 0.00 | 0.00 | 0.00 | 0.00 | 0.00 |
|  | **Year** | 0.54 | 0.09 | 0.20 | 0.54 | 0.94 | 0.87 |
|  | **Season** | 0.00 | 0.01 | 0.00 | 0.00 | 0.00 | 0.00 |
|  | **Size of the fish** | 0.03 | 0.35 | 0.30 | 0.16 | 0.00 | 0.00 |
| **Interaction** | **Year·Season** | 0.69 | 0.22 | 0.93 | 0.67 | 0.85 | 0.37 |
|  | **Year·Size** | 0.78 | 0.48 | 0.42 | 0.33 | 0.91 | 0.23 |
|  | **Season·Size** | 0.29 | 0.74 | 0.64 | 0.47 | 0.10 | 0.22 |
|  | **Year·Season·Size** | 0.30 | 0.05 | 0.39 | 0.56 | 0.45 | 0.90 |
